# Oxygen in Therapeutics

**Published:** 1887-03

**Authors:** Charles J. Smith


### CULLINGS FROM CONTEMPORARIES.

### OXYGEN IN THERAPEUTICS.

*By Charles J. Smith, M. R. C. S., F. R. G. S., &c.*

ENGLISH medical literature is singularly deficient in works upon the value of oxygen as an addition to our therapeutic agents—and for this reason, that from one cause or another, but chiefly from the difficulty and cost of production of the gas in any quantity, oxygen has never been brought into general use by the profession in this country, so that no large experience of its advantages has been gained; while, unfortunately, those persons who have written at all upon the subject have done so in such a manner as to have exposed it to the suspicion of quackery. This is greatly to be deplored, because there can be no question that the gas which Lavoisier characterized as "the vivifying spirit *par excellence*" is an agent of the highest value to the physician.

Those who are desirous of learning what has been done with oxygen must look to the papers read before the various scientific societies on the Continent, and to the records of the practice of many distinguished continental physicians. Demarquay appears to have been a somewhat enthusiastic believer in the efficacy of oxygen inhalation, and he has written strongly upon the subject in his work on "Pneumo-therapeutics." He considers this as one of the safest agents in the treatment of pulmonary phthisis in every stage of the disease, and that its curative action is still more manifest in bronchitis, asthma (spasmodic no doubt is meant), and whooping-cough. Hayem, as well as Demarquay, reports favorably of its use in anæmia and scrofula. Durand, Fardel, Béranger, Feraud, and Thierry, Mieg have utilized its combustible action in the treatment of diabetes, in which disease its use is highly extolled by Dr. E. Morin, who, in his prize essay, read before the Society of Medicine in Antwerp, states: "In inhaling from ten to twenty litres of oxygen every morning the sufferer from diabetes will add to his treatment an incontestably useful agent, especially if his lungs have any tendency to become congested." Dr. Campardon read a paper before the Society of Practical Medicine in

Paris. Mayer, Maunoir, Pinard, and Dorean have published among them the details of eight cases, of which the last three were cases of incessant vomiting. Quinquod and Kirnberger have also contributed to the records of success which has attended the administration of oxygen. The most recent, and perhaps the most interesting, use made of this agent was during the late terrible epidemic of cholera at Toulon and Marseilles, when highly favorable results were obtained, and are duly recorded in the official reports of the Chief Medical Officer of the Marine. There is every reason to believe that the surgeon, too, may find a faithful aid in oxygen. Langier appears to have met with considerable success in the treatment of senile gangrene and "local asphyxia congestion" by oxygenated baths. There are many others who have published the results of their experience with oxygen; but those already quoted will be sufficient to show how well worth attention it is as a therapeutic agent.

The two great difficulties which have opposed the use of oxygen on the very threshold, have been the cost of its production and its impurity when produced. These difficuties, however, have been successfully overcome by MM. Brin, members of the Societété d'Hygiéne Francais, who have succeeded (after the devotion of fifteen years of their lives to the subject) in perfecting a process by which oxygen is obtained in an absolutely pure state from atmospheric air, and this at a cost which places it as much within reach as the ordinary medicinal agents in daily use. This process of obtaining oxygen is really a most simple one. Although Boussingault stated in a report made to the French Academy of Science thirty years ago that oxide of barium was an unstable agent for the purpose, yet it is this selfsame agent that MM. Brin have succeeded in making their servant. Their anhydrous oxide of barium, placed in retorts heated to a given temperature, and served with a supply of purified atmospheric air, will give off every two hours oxygen at the rate of one cubic foot per pound of barium oxide. Perhaps the most interesting point in connection with this process is that it is absolutely a mechanical one. There is no loss or waste of the agent employed—namely, the anhydrous oxide of barium. There is no visible difference under the microscope between the low and the peroxide, and no chemical change takes place. The oxygen is absorbed from the purified air passed through the retorts under pressure; it is given off again under vacuum; and the proof that in carrying out this method MM. Brin call to their aid no complex chemical decomposition is, that the same pound of barium, increased in weight when it takes up the oxygen, returns to its exact original weight when, under vacuum, the oxygen is yielded up. Moreover this same pound of barium is at once ready to perform, and repeat *ad infinitum*, its task of absorbing and yielding up this perfectly pure oxygen.—*Lancet*.

From the tenor of Dr. Smith's paper it might be inferred that nothing had been done, on this side the Atlantic, toward the investigation of oxygen therapeutics; whereas, the fact is that Europeans might learn something from American experience in connection with this agent.

The quackery associated with the subject, and in this line America deserves the palm—has nothing to do with its legitimate study. As long ago as 1869 a suggestive papcr appeared in the *Chicago Medical Journal*, and in 1870, Dr. Andrew H. Smith of New York read a prize essay before the Alumni Association of the College of Physicians and Surgeons, which gave a very fair resumé of the history and uses of oxygen gas.

During the past five years numerous papers on the subject have appeared in the medical press of this country, those of Dr. Samuel S. Wallton of New York, being most complete and voluminous.

According to the latter a considerable success has followed the judicious use of oxygen and allied gases in the indigestions and in many forms of bronchial and pulmonary affections; while contrary to the assertions of the English authority above quoted, no very complex apparatus is required, nor is it at all difficult to prepare a perfectly reliable quality of gas for medicinal use.

We hope practitioners on this side will continue their experimentation, and add to the rather meager data thus far available.

